# 

**Published:** 1889-09

**Authors:** 


THE Southern Surgical and Gynecological Association will hold its next annual session at Nashville, Tennessee, November 12, 13 and 14. Hunter McGuire, M. D., LL. D., is president, and W. E. B. Davis, M. D., secretary. A cordial invitation to attend is extended to the profession. A long list of papers is announced to be read on the occasion.

A SAVAGE.—A man, on entering an omnibus, came near falling, and to steady himself caught at the nearest object, which was a lady's lap.

"You are a regular savage, Sir!" she said.

"Yes'm; of the paw-knee tribe," he meekly admitted.

